# Psychological correlates of adherence to photoprotection in a rare disease: International survey of people with Xeroderma Pigmentosum

**DOI:** 10.1111/bjhp.12375

**Published:** 2019-06-10

**Authors:** Jessica Walburn, Martha Canfield, Sam Norton, Kirby Sainsbury, Vera Araújo‐Soares, Lesley Foster, Mark Berneburg, Alain Sarasin, Natalie Morrison‐Bowen, Falko F. Sniehotta, Robert Sarkany, John Weinman

**Affiliations:** ^1^ School of Cancer and Pharmaceutical Sciences, Faculty of Life Sciences and Medicine King’s College London UK; ^2^ Health Psychology Section, Institute of Psychiatry, Psychology and Neuroscience King’s College London UK; ^3^ Department of Inflammation Biology, Faculty of Life Sciences and Medicine King’s College London UK; ^4^ Faculty of Medical Sciences, Institute of Health and Society Newcastle University UK; ^5^ National Xeroderma Pigmentosum Service Guy’s and St. Thomas’ NHS Foundation Trust London UK; ^6^ Department of Dermatology Universitätsklinikum Regensburg Germany; ^7^ Institute of Cancer and Genetics Gustave Roussy Institute, UMR8200 CNRS Villejuif France; ^8^ School of Medicine University of Leeds UK

**Keywords:** adherence, illness perceptions, photoprotection, treatment beliefs, Xeroderma Pigmentosum

## Abstract

**Objectives:**

Xeroderma pigmentosum (XP) is an extremely rare genetic disorder (approximately 100 known cases in the United Kingdom), where DNA damage caused by ultraviolet radiation in daylight cannot be repaired. Adherence to photoprotection is essential to prevent skin cancer. We investigated psychological correlates of photoprotection in the XP population of Western Europe and the United States.

**Design:**

Cross‐sectional survey of adults with XP and caregivers of patients <16 years and those with cognitive impairment in the United Kingdom, Germany, the United States, and France (*n* = 156).

**Methods:**

Photoprotection activities to protect the face and body when outdoors; avoidance of going outside during daylight hours; intention; self‐efficacy; and social support were assessed using measures developed for this study. Participants answered questions about their illness representations of XP (BIPQ); beliefs about photoprotection (BMQ); automaticity (i.e., without conscious effort) (SRBAI); clinical and demographic characteristics. Ordinal logistic regressions determined factors associated with photoprotection.

**Results:**

One third did not achieve optimal face photoprotection. After controlling for demographic and clinical factors, modifiable correlates of higher photoprotection included greater perceived control of XP, stronger beliefs in necessity and effectiveness of photoprotection, and higher intention. Avoidance of going outside was associated with greater photoprotection concerns, more serious illness consequences, and higher XP‐related distress. Greater automaticity and higher self‐efficacy were associated with better protection across all outcomes.

**Conclusions:**

Approximately half of all known cases across three European countries participated. Identified modifiable predictors of photoprotection may be targeted by interventions to reduce the incidence of skin cancers in the immediate future, when a treatment breakthrough is unlikely.

**Statement of contribution**

***What is already known on this subject?***

- Adherence to photoprotection in other populations at elevated risk from skin cancer is poor; however, the level in XP is unknown.
- Research across chronic conditions shows that adherence to treatment and lifestyle recommendations are influenced by illness perceptions, self‐efficacy, and treatment beliefs.
- Studies on photoprotection conducted with the general population have found that perceived risk, perceptions of ultraviolet radiation (UVR) protection, self‐efficacy for the behaviour, and automaticity (behaviours that are enacted with little conscious awareness) are related to better photoprotection.

***What does this study add?***

- This is the first international survey to examine adherence and its correlates in people with XP (an under‐researched group at very high risk of fatal skin cancer). Adherence varies and at least one third have potential for improvement.
- Perceptions about XP, photoprotection beliefs, self‐efficacy, intention, and automaticity were associated with photoprotection of the face and body when outdoors.
- Negative emotional representations of XP were associated with avoidance of going outside during daylight hours.

## Background

Xeroderma pigmentosum (XP) is a rare autosomal recessive genetic disorder with an incidence of 2.3 per million live births in Western Europe (Kleijer *et al*., 2008), ~100 cases in the United Kingdom. Individuals cannot repair damage to DNA caused by ultraviolet radiation (UVR) in daylight. Xeroderma pigmentosum can be broken down into eight different subtypes, known as complementation groups (A, B, C, D, E, F, G, and V), corresponding to the eight affected genes involved in the UVR repair pathway (Lehmann, McGibbon, & Stefanini, 2011). Patients develop skin cancers, often from early childhood, eye disease, and around 25% of patients have fatal neurological degeneration. The median lifespan is 32 years, with 60% of premature deaths due to malignant melanoma skin cancer (Bradford *et al*., 2011). The only way to improve the prognosis is through extremely rigorous protection against UVR. The aim is to keep UVR exposure to the absolute minimum, as there is no known ‘safe’ dose. This is a major burden on patients and their families, with optimal photoprotection involving UVR‐protective face visors, wearing gloves, hats, and sunscreen, and avoidance of daylight (Tamura, DiGiovanna, Khan, & Kraemer, 2014).

Despite the importance of photoprotection and the taxing nature of practicing daily protection, there has been no empirical estimation of levels of adherence to photoprotection in XP. An N‐of‐1 study of our sample showed that adherence varied between and within individuals (Sainsbury *et al.,* 2018 ) and a qualitative analysis explained this in terms of differences in individuals’ perceptions of the necessity of photoprotection and its psychosocial impacts in terms of appearance, self‐identity, stigma, and activity restrictions (Morgan *et al.,* 2019). Adherence is also poor in non‐XP survivors of malignant melanoma (Nahar *et al*., 2016). In XP, where the risk of melanoma is 2,000‐fold greater than the general population (Bradford *et al*., 2011), significant non‐adherence to photoprotection substantially increases the risk for morbidity and mortality.

For patients who are non‐adherent, the design of effective behaviour change interventions is required and needs to be informed by knowledge of psychological factors associated with poor protection. In the general population, photoprotection behaviour has been associated with beliefs about photoprotection [(e.g., personal vulnerability, benefits of protection, barriers (Bränström *et al*., 2010; Pettigrew *et al*., 2016)], self‐efficacy (Good & Abraham, 2011), intention, and automaticity (behaviours that are enacted with little conscious awareness*)* (Allom, Mullan, & Sebastian, 2013). The importance of treatment beliefs, and to a lesser extent illness perceptions, in explaining adherence has been reported across other chronic conditions (e.g., Broadbent, Donkin, & Stroh, 2011; Horne *et al*., 2013). The related qualitative studies (Anderson, Walburn, & Morgan, 2017; Morgan *et al.,* 2019) identified a range of determinants (e.g., emotional distress, appearance concerns, perceived social support), and we wished to explore whether these associations would be present in a larger representative sample.

The aim of this study was to identify modifiable psychological factors related to photoprotection activities in XP to inform the development of an intervention designed to improve photoprotection. This is of considerable importance in XP, where quick advances in medical treatments are unlikely due to lack of funding (Oo & Rusch, 2016). Interventions showing improvements in photoprotection among individuals at elevated risk for melanoma highlight the potential for behaviour change (Wu *et al*., 2016 – for a systematic review). Approaches to complex intervention design such as the Behaviour Change Wheel (Michie, van Stralen, & West, 2011) and Intervention Mapping (Eldredge, Markham, Kok, Ruiter, & Parcel, 2016) are theory agnostic, and recommend using broad frameworks based on multiple theories such as Theoretical Domains Framework (TDF) (Araújo‐Soares, Hankonen, Presseau, Rodrigues, & Sniehotta, 2019). Given that this study is part of formative research informing an intervention for a complex set of behaviours and that recent reviews of adherence interventions conclude that no single theoretical model sufficiently incorporates all known determinants; >700 identified by a review of reviews (Kardas, Lewek, & Matyjaszczyk, 2013), we selected variables on the basis of the TDF (Cane, O’Connor, & Michie, 2012) and the updated Common‐Sense Model of self‐regulation (CSM) (Leventhal, Phillips, & Burns, 2016). Given that this was the first survey of psychosocial correlates of adherence in XP, we added variables that the Patient and Public Involvement (PPI) panel and clinical stakeholders considered to be important (e.g., concerns about the impact of photoprotection on family and friends) or had emerged within early qualitative interviews (e.g., identity and appearance concerns, social stigma, and role of social support).

The objectives of the study were threefold: (1) identify levels of adherence to photoprotection recommendations in XP; (2) describe the beliefs about the condition and recommended photoprotection regime of people diagnosed with XP; and (3) identify potentially modifiable psychological factors that might constitute intervention targets.

## Methods

### Design and participants

This study is part of a mixed‐methods programme of research aimed at improving photoprotection practices in XP patients (Walburn *et al*., 2017). The cross‐sectional survey was completed between May and December 2016 by 156 participants in the United Kingdom, France, Germany, and the United States. The target sample size was set at 193, which represents approximately 80% of known cases in the United Kingdom, France, and Germany. This target sample size would have allowed detection of correlations 0.2 (80% power; 5% alpha) and, in hierarchical ordinal logistic regression models, psychological variables that explained at least an additional 5% of the variance (i.e., adjusted odds ratio’s >2.4) after initially controlling for clinical and demographic factors (>90% power; 5% alpha). Due to lower than anticipated recruitment rates, additional patients were recruited from the United States. The overall recruitment rate was 57.3% of those invited (the United Kingdom 84.6%, France 70.7%, Germany 72%, and the United States 40.5%). It is important to accentuate that even though the sample size of 156 participants is small, it represents approximately half of known cases across three countries in Western Europe, mitigating this weakness.

### Procedure

In the United Kingdom and Germany, people diagnosed with XP were invited to participate by research staff working in the Specialist XP Clinics in London and Regensburg, respectively. Participants were sent an invitation letter and information sheet or, if they were due to attend the clinic, were approached in person. In France and the United States, patients were invited to participate via patient support groups by post or at scheduled events. Patients were from several regional areas within the countries. All participants over the age of 16 years gave fully informed consent, apart from in the United Kingdom where due to REC requirements we also obtained assent (<18 years). For patients under the age of 16 and/or people with cognitive impairment, we obtained consent from their parent or caregiver. The study was approved by the relevant ethical and regulatory bodies (the  UK: London – Camden & Kings Cross Research Ethics Committee 15/LO/1355 (FRA: Authorization was given by the Agence de Biomédecine and by the Commission Nationale de l’ Informatique et des Libertés to Alain Sarasin; GER: Approved by the local ethics committee at the Universitätsklinikum Regensburg; the USA: Permission was covered by existing data protection legislation). Adult patients without cognitive impairment completed the survey without assistance. For all children (<16 years) and adults with significant cognitive impairment, the patient’s primary caregiver completed the survey about the patient, referred to as ‘the cared‐for sample’. The term ‘adult sample’ refers to patients aged 16 years or above without cognitive impairment.

### Measures

Two versions of the survey were devised: one for the adult sample and one for the cared‐for sample. The cared‐for sample version assessed identical determinants of photoprotection to the adult version, the difference being in the phrasing of the question, framed in relation to the caregiver (e.g., *How often do you/does he or she wear a face visor? How much do you think XP treatment in the clinic (e.g., surgery, creams) can help your/their skin or eye health?)*. The survey was developed in English and translated to French and German using forward and backward translation to achieve equivalence of meaning (WHO, 2018).

#### Photoprotection activities

Due to the extreme nature of protection required in XP, and lack of an appropriate existing questionnaire, a bespoke assessment of photoprotection was developed for the study (Canfield *et al*., 2018).

The questionnaire comprised two subscales: a fourteen‐item subscale about adherence to photoprotection to the face (seven items regarding cloudy days and seven items regarding sunny days) and a ten‐item subscale about adherence to photoprotection to the body (five items for each of cloudy and sunny days). Participants are asked to report how often, whilst outside, in the last seven days they wore/used: a face visor, hat, glasses, sunscreen on the face, on the arms/hands and legs, lips sunscreen, scarf or face‐buff, hoodie (worn up), long sleeves, gloves, and long trousers/thick tights. Responses ranged from 1 (Never) to 5 (Always). A framework for scoring adherence to face photoprotection was created to avoid penalizing one photoprotection activity over another (e.g., if wearing a face visor, there is no benefit from wearing a scarf underneath). The seven individual behaviours associated with face photoprotection were combined and reduced into five observed scores based on regions of the face: forehead (face visor, hoodie, face sunscreen), lower face (face visor, scarf, lips sunscreen), nose (face visor, face sunscreen), cheeks and sides (face visor, face sunscreen), and eyes (face visor, sunglasses) for both cloudy and sunny days. Similarly, adherence to body photoprotection behaviours was defined by the sum of two body areas: arms (long sleeves and sunscreen on the arms/hands) and legs (long trousers and sunscreen on the legs) for both cloudy and sunny days. Adherence to face/body photoprotection behaviours was defined by the sum of the areas and an average score between both cloudy and sunny days. The maximum score for each scale was 5, indicating optimal photoprotection. No safe level of UVR exposure for people diagnosed with XP has been identified; therefore, any score below 4 (indicating moderate to no photoprotection) is interpreted as non‐adherent to recommendations. Internal reliability assessed using Cronbach’s alpha, for both face and body scales, was high (α = .93 and α = .88, respectively). For the UK sample (not assessed in other countries), adherence to face photoprotection correlated highly with an objective measure of average daily facial photoprotection (*r* = .66). For further information about the development and validation process of this measure, see (Canfield *et al*., 2018).

The extent to which participants avoided going outside during the day was measured separately on two Likert scale items, one for cloudy days and one for sunny days, ranging from 1 (Never) to 5 (Always). The total score for avoidance of going outside was defined as the average of the two items.

#### Potential correlates of photoprotection activities

##### Demographics

Information about gender, age, and education level (ranging from 0, no qualification, to 5, postgraduate degree), was collected.

##### Clinical characteristics

Respondents were asked their age at the time of diagnosis, whether they ever had a diagnosis of any skin cancer, neurological manifestations of the XP (hearing, walking, cognition, or speaking), eye disease, and XP genetic complementation group. Since some patients experience an enhanced sunburn response, they were classified as ‘burners’ if they responded positively to at least two of three items regarding how easily they sunburn (*e.g., Have you ever had sunburn so badly you needed to see a doctor about it*? *Yes/no*) (Sethi *et al*., 2013).

#### Psychological characteristics

##### Perceptions of photoprotection

Perceptions relating to the need for photoprotection (an umbrella term for all protection activities) were measured using a modified version of the Beliefs about Medicines Questionnaire (BMQ) (Horne, Weinman, & Hankins, 1999) necessity and concerns subscales. Each item is scored on a five‐point scale *from 1 (Strongly disagree) to 5 (Strongly agree). The* average score across items in each subscale was used in the analysis. Cronbach’s alpha was .73 and .80 for necessity and concerns, respectively.

##### Effectiveness of photoprotection behaviours

Participants were asked to report to what extent they believed their activities had effectively protected against UVR in the last 7 days *(Thinking about all the things you did to protect yourself over the past 7 days (e.g., wearing sunscreen, wearing a hat), how well do you think they protected you from UVR?)*. Responses ranged from 1 (*Not at all*) to 5 (*Completely*).

##### Beliefs about XP

An adapted version of the nine‐item Brief Illness Perception Questionnaire (BIPQ) (Broadbent, Petrie, Main, & Weinman, 2006) was used to assess the following dimensions of patients’ perceptions of their XP: consequences, timeline, personal control of XP, photoprotection control of XP, treatment control, identity, negative emotional representation, and perceived understanding, using single items. Each item is scored on an 11‐point scale (0–10), control and perceived understanding items are reverse scored, with higher scores representing a stronger and more negative perception of each specific dimension.

##### Intention to photoprotect

A 10‐item questionnaire was designed to measure intention (motivation) to engage in photoprotection activities when outside. Participants were asked to report their level of intention for each type of photoprotective activity in the next 7 days (wearing a face visor; hat; glasses; sunscreen; lip sunblock; hoodie; long sleeves; gloves; long trousers/thick tights) separately (e.g., *I intend to protect myself by wearing a face visor*). Responses range from 1 (*Strongly disagree*) to 7 (*Strongly agree*). The mean score across items was used in the analysis (Cronbach’s alpha, α = .73). Intention to avoid going outside during the daytime in the next 7 days was assessed with a single item (*I intend to protect myself by avoiding going outside in the daytime)*. Responses range from 1 (‘Strongly disagree’) to 7 (‘Strongly agree’). To facilitate respondent completion of the questionnaire, the format of these and the self‐efficacy items (see below) were adapted from a manual for designing questionnaires based on the Theory of Planned Behaviour (Francis *et al*., 2004), which distils current evidence on how best to operationalize intention and perceived behavioural control which is conceptually similar to self‐efficacy.

##### Self‐efficacy for photoprotection activities

Confidence to protect against UVR was assessed by 10 items using a similar structure. Participants were asked to report how confident they were that they would be able to carry out each photoprotection activity over the next 7 days (e.g., *When I am outside in the next 7 days I am confident I could wear a face visor*) using a Likert scale ranging from 1 (*‘Strongly disagree’*) to 7 (*‘Strongly agree’*). The mean score across items was used in the analysis (Cronbach’s alpha, α = .75). Confidence to avoid going outside during the daytime in the next 7 days was assessed using the same Likert scale.

##### Automaticity of photoprotection activities

Participants were asked to rate the extent to which they thought that they were carrying out each photoprotection activity automatically, every time they were ready to go outside over the last 7 days *(e.g., Wearing a hat was something I did automatically without thinking)*. Responses range from 1 (*Strongly disagree that the behaviour was automatic*) to 7 (*Strongly agree that the behaviour was automatic*). The item stem was adapted from the Self‐Report Behavioural Automaticity Index (SRBAI) (Gardner, Abraham, Lally, & de Bruijn, 2012) a subscale from the Self‐Report Habit Index (Verplanken & Orbell, 2003) and selected as index of automaticity (B. Gardner, personal communication, 17 November 2016). The mean score across items was used in the analysis (Cronbach’s alpha, α = 0.71). The extent to which participants avoided going outside automatically in the previous 7 days was assessed using the single item (*Avoiding going outside during the day was something I did automatically without thinking)* with the same 1–7 Likert scale.

##### Perceived social support

Level of perceived support with UVR protection was measured by the mean of two items: amount *(How much support or help do you have from the people around you with your UV protection?)* and quality of support *(How satisfied are you with the support or help that you have to help you with your UV protection?*). These single items represented the two dimensions of support (level and degree of satisfaction) from the Social Support Questionnaire (SSQ) (Sarason, Levine, Basham, & Sarason, 1983). Responses ranged from 1 (*No support*) to 5 (*Comprehensive support*).

#### Analysis

Descriptive statistics were calculated using frequencies and percentages for categorical data, and means and standard deviations for continuous data. The association between psychological variables with adherence to face photoprotection, body photoprotection, and avoidance of going outside was examined in univariate ordinal logistic regressions. Variables with standardized odds ratios ≥ 1.40 (a small effect, equivalent to a standardized regression coefficient of .1) on at least one photoprotection outcome (adherence to face photoprotection; adherence to body photoprotection; avoidance of going outside) in the univariate analyses were entered in ordinal logistic regressions to ascertain the strength of associations with photoprotection outcomes when adjusted for demographic (age, gender, country, skin colour) and clinical variables (age at time of diagnosis, history of skin cancer, and burn status). The amount of variance explained by demographic, clinical, and potentially modifiable psychological variables in each photoprotection outcome was assessed in a series of hierarchical ordinal logistic regressions. In each case, the demographic and clinical variables were entered in the first step and all psychological variables were entered in the second step.

## Results

### Adherence to photoprotection recommendations

Table 1 presents the sample characteristics for the total sample (*N* = 156). Using the total score, photoprotection adherence was higher for the body (*M* = 4.2 out of 5; *SD* = 1.0) than for the face (*M* = 3.7; *SD* = 1.2). The mean score for avoidance of going outside was *M* = 2.9 (*SD* = 1.2). There was a strong association between face and body photoprotection (*r *= .77) but weak associations with either face or body photoprotection and avoidance of going outside (*r* = .14 and *r* = .06, respectively).

**Table 1:** Demographic, clinical, psychological, and photoprotection characteristics of the sample

	Total (*N* = 156)	Adult patient sample (*n* = 71)	Cared‐for sample (caregivers) (*N* = 85)	*p* (adult patient vs. caregivers)
Demographic variables				
Male, *n* (%)	79 (50.6%)	37 (52.1%)	42 (49.4%)	.737
Age, mean (*SD*)	24.81 (19.46)	39.03 (19.23)	12.94 (8.76)	<.001
Skin colour (brown/black = 0), *n* (%)	100 (64.5%)	44 (62%)	56 (66.7%)	.543
Education level, *n* (%)				
No qualifications	26 (26.0)	6 (8.7)	20 (64.5%)	<.001
Secondary school	12(12.0)	9 (13.0)	3 (9.7%)	
Post‐school qualification	24 (24.0)	19 (27.5)	5 (16.1%)	
Professional qualifications	24 (24.0)	22 (31.9)	2 (6.5%)	
Higher education diploma	14 (14.0)	13 (18. 1)	1 (3.2%)	
Country, *n* (%)				
UK	66 (42.3)	39 (54.9)	27 (31.8%)	.021
France	58 (37.2)	22 (31.0)	36 (42.4%)	
Germany	15 (9.6)	6 (8.5)	9 (10.6%)	
USA	17 (10.9)	4 (5.6)	13 (15.8%)	
Clinical variables				
Age at the time of diagnosis, *mean* (*SD*)	10.94 (14.73	19.18 (17.74)	4.09 (5.80)	
Burners, *n* (%)	47 (30.1)	18 (25.4)	29 (34.1%)	.235
Skin cancer, *n* (%)	71 (45.2)	45 (63.4)	25 (29.4%)	<.001
XP causing cognitive problems, *n* (%)	35 (22.4)	8 (11.3)	26 (31.0%)	
Eyes problems, *n* (%)	112 (71.8)	50 (70.4)	62 (72.9%)	.728
Psychological variables, mean (*SD*)				
Illness perception (0–10)				
Consequences	7.24 (2.66)	6.54 (2.85)	7.82 (2.36)	.002
Timeline	9.46 (1.69)	9.75 (1.26)	9.22 (1.97)	.055
Personal control of XP	6.05 (3.07)	5.89 (2.73)	6.19 (3.34)	.544
Photoprotection control of XP	8.87 (1.85)	8.15 (2.28)	9.47 (1.09)	<.001
Treatment control	8.15 (2.20)	8.29 (1.99)	8.02 (2.37)	.444
Identity	5.74 (3.08)	5.54 (3.13)	5.90 (3.06)	.457
Illness concern	6.44 (3.09)	6.48 (3.08)	6.41 (3.12)	.893
Understanding	7.14 (2.76)	8.25 (1.73)	6.21 (3.10)	<.001
Emotional representation	6.06 (3.24)	5.51 (3.45)	6.53 (3.00)	.049
Beliefs about photoprotection (1–5)				
Necessity	4.41 (0.72)	4.11 (0.80)	4.66 (0.52)	<.001
Concern	2.98 (0.99)	2.79 (0.95)	3.15 (0.99)	.024
Intention to photoprotect (1–7)	5.10 (1.19)	4.83 (1.21))	5.34 (1.12)	.010
Intention to avoid going outside	4.34 (2.37)	4.51 (2.39)	4.20 (2.37)	.428
Self‐efficacy to photoprotect^a^ (1–7)	5.20 (1.21)	4.01 (1.21)	5.52 (1.13)	.001
Self‐efficacy to avoid going outside	4.13 (2.38)	3.93 (2.31)	4.30 (2.45)	.342
Automaticity of photoprotection^a^ (1–7)	4.44 (1.42)	4.01 (1.39)	4.79 (1.34)	.001
Automaticity of avoidance of going outside	4.49 (2.83)	4.23 (2.84)	4.71 (2.82)	.293
Social support (1–5)	3.70 (1.16	3.60 (1.02)	3.33 (1.20)	.150
Effectiveness of photoprotection^a^ (1–5)	3.75 (1.00)	3.47 (.92)	3.96 (1.02)	.002
Photoprotection behaviours, mean (*SD*)				
Adherence to face photoprotection practices^a^	3.69 (1.17)	2.89 (1.22)	4.19 (.73)	<.001
Adherence to body photoprotection practices^a^	4.21 (.99)	3.70 (1.10)	4.65 (.63)	<.001
Avoid going outside	2.92 (1.21)	3.05 (1.16)	2.82 (1.24)	.234

Note

a Wearing sunscreen, clothing when outside.

One third (35.3%) reported suboptimal adherence to face photoprotection. Face photoprotection was higher on sunny (*M* = 4.27, *SD* = 1.04) compared to cloudy days, *M* = 4.01, *SD* = 1.28, *F*(4, 149) = 140.5, *p* < .001, with the largest weather‐dependent difference for sunscreen use (47.4% used on cloudy, 59.6% sunny days). The cared‐for sample was better protected than the adults on the face (*M* = 4.19, *SD* = 0.73 vs. *M* = 2.89, *SD* = 1.22) and body (*M* = 4.65, *SD* = 0.63 vs. *M* = 3.70, *SD* = 1.10). A minority of adults wore a visor (32.4%), whereas a large proportion of the cared‐for sample used it on sunny days (85.9%).

### Psychological characteristics of the sample

The psychological characteristics are reported in Table 1. The BIPQ scores showed that participants perceived XP to have serious consequences, to be a chronic condition that could be effectively managed by treatment, and with a moderate negative emotional response. Overall, current photoprotection was perceived to be an effective barrier from UVR, with 66.2% reporting they were ‘completely’ or ‘very well’ protected. Beliefs about the necessity of photoprotection were high (*M* = 4.41 out of 5, *SD* = 0.72), and there was less concern about having to photoprotect (*M* = 2.98, *SD* = 0.99). Participants reported strong intention to photoprotect (*M* = 5.10 out of 7, *SD* = 1.19) and were generally confident that they could carry out photoprotection (*M* = 5.20, *SD* = 1.21).

Compared to adult patients, caregivers perceived XP to be more serious, were more convinced that photoprotection could control the condition, reported having a lower understanding of XP, and felt it had a greater negative emotional impact on the patient. Caregivers thought protection was more necessary and effective, although had more concerns. Caregivers also reported stronger intention to protect the person they were caring for, higher self‐efficacy, and greater automaticity than adults.

### Factors associated with photoprotection activities

Univariate results can be found in the Table S1. Considering only the adult sample, younger chronological age and younger age at diagnosis were associated with increased photoprotection to the face (OR = .61, *p* < .05; OR = .59, *p* < .05) and body (OR = .68, *p* < .05; OR = .74, *p* < .05). Education (adult patients only) was not associated with photoprotection to the face or body. Clinical factors, including higher propensity to burn and prior skin cancer, were not significantly related to photoprotection when outdoors or avoidance of going outdoors.

After controlling for demographic and clinic factors (Table 2), perceptions of greater personal control over the health impact of XP (OR 1.72, 95% CI 1.20, 2.45; OR 1.63, 95% CI 1.15, 2.30), greater perceived photoprotection control of XP (OR 1.64, 95% CI 1.19, 2.26; OR 1.39, 95% CI 1.02, 1.90), stronger belief in the necessity of photoprotection (OR 1.88, 95% CI 1.34, 2.64; OR 2.09, 95% CI 1.46, 2.99), and effectiveness of protection against UVR (OR 2.22, 95% CI 1.53, 3.24; OR 2.09, 95% CI 1.39, 2.76), and greater intention to photoprotect (OR 1.83, 95% CI 1.28, 2.61; OR 1.59, 95% CI 1.14, 2.20) were significantly associated with higher adherence to face and body photoprotection but not related to avoidance of going outside. In contrast, higher XP‐related distress (OR 2.11, 95% CI 1.54, 2.89) and concern (OR 1.65, 95% CI 1.20, 2.25), stronger belief that XP has serious consequences (OR, 1.47, 95% CI 1.09, 1.99) and greater concerns about protecting against UVR (OR 1.80, 95% CI 1.32, 2.46),were all significantly associated with avoidance of going outside, but not related to adherence to face or body photoprotection (except consequences for the latter). Self‐efficacy was related to all photoprotection outcomes (OR 1.83, 95% CI 1.28, 2.61; OR 1.68 , 95% CI 1.20, 2.12; OR 2.20, 95% CI 1.61, 3.00; adherence to face and body photoprotection and avoidance of going outside, respectively) as was automaticity (OR 2.16, 95% CI 1.47, 3.19; OR 2.20, 95% CI 1.52, 3.18; OR 2.52, 95% CI 1.82, 3.49; adherence to face and body photoprotection and avoidance of going outside, respectively).

**Table 2:** Multivariable logistic regression of photoprotection activities on psychological variables adjusted for demographic and clinical factors

	Adherence to face photoprotection OR (95% CI)	*p*	Adherence to body photoprotection OR (95% CI)	*p*	Avoidance of going outside OR (95% CI)	*p*
XP Illness perception						
Consequences	1.25 (0.86, 1.81)	.238	**1.42 (1.01, 1.99)**	**.038**	**2.11 (1.54, 2.89)**	**<.001**
Personal control of XP	**1.72 (1.20, 2.45)**	**.003**	**1.63 (1.15, 2.30)**	**.005**	0.77 (0.56, 1.05)	.097
Photoprotection control of XP	**1.64 (1.19, 2.26)**	**.002**	**1.39 (1.02, 1.90)**	**.036**	0.99 (0.73, 1.34)	.953
Illness concern	0.96 (0.66, 1.39)	.830	1.10 (0.79, 1.55)	.555	**1.65 (1.20, 2.25)**	**.002**
Understanding	0.92 (0.81, 1.05)	.223	0.90 (0.80, 1.02)	.110	0.94 (0.84, 1.09)	.262
Emotional representation	1.24 (0.88, 1.75)	.219	1.32 (0.95, 1.84)	.098	**1.47 (1.09, 1.99)**	**.011**
Beliefs about photoprotection						
Necessity	**1.88 (1.34, 2.64)**	**<.001**	**2.09 (1.46, 2.99)**	**<.001**	1.33 (0.98, 1.81)	.066
Concern	1.14 (0.80, 1.64)	.455	1.05 (0.65, 1.70)	.846	**1.80 (1.32, 2.46)**	**<.001**
Intention to photoprotect^a^/avoid going out	**1.83 (1.28, 2.61)**	**.001**	**1.59 (1.14, 2.20)**	**.005**	1.09 (0.86, 1.37)	.483
Self‐efficacy of photoprotection^a^/avoid going out	**1.83 (1.29, 2.60)**	**.001**	**1.68 (1.20, 2.12)**	**.002**	**2.20 (1.61, 3.00)**	**<.001**
Automaticity of photoprotection^a^/avoid going out	**2.16 (1.47, 3.19)**	**<.001**	**2.20 (1.52, 3.18)**	**<.001**	**2.52 (1.82, 3.49)**	**<.001**
Effectiveness of photoprotection^a^	**2.22 (1.53, 3.24)**	**<.001**	**1.96 (1.39, 2.76)**	**<.001**	1.10 (0.82, 1.47)	.531
*R* ^2^ model for demographic and clinical variables	.08		.05		.01	
*R* ^2^ model for demographic, clinical and psychological variables						
	.22		.22		.06	

Notes

Models adjusted for gender, age, skin type, country, age at time of diagnosis, burning type, and history of skin cancer. Bold indicates statistically significant associations.

a Wearing sunscreen, clothing when outside.

Hierarchical multivariable ordinal logistic regressions examined the amount of variance explained by all potentially modifiable psychological factors identified in the previous analysis (Table 2). In the first step, demographic and clinical variables explained between 1 and 8% of variance in photoprotection behaviour. Psychological variables, entered in the second step, explained an additional 5% of the variance in avoidance of going outside, 14% in adherence to face photoprotection, and 17% in adherence to body photoprotection (all *p* < .05).

## Discussion

This is the first survey of adherence to photoprotection to be conducted in people living with XP. Reported adherence to photoprotection was suboptimal for around one third of individuals. Stronger perceptions of the extent to which photoprotection can control the health consequences of XP, stronger beliefs about the necessity of protecting, higher intention, self‐efficacy, and automaticity were related to better photoprotection whilst outside. Avoiding exposure by staying indoors was associated with a different pattern of predictors, with negative emotional representations and concerns about XP and photoprotection being more important. The range of factors supports the use of unified frameworks and models that include a wider variety of variables, such as the recent extension to the CSM by Hagger and colleagues to incorporate attitudes, self‐efficacy, intentions, and action plans (Hagger, Koch, Chatzisarantis, & Orbell, 2017).

Automaticity, a feature of but not limited to habitual behaviour (Marteau, Hollands, & Fletcher, 2012), was an important determinant for better photoprotection outdoors and avoiding daylight altogether. These findings support the targeting of automatic alongside deliberative processes and are consistent with phase models of behaviour, which include volitional and motivational constructs [e.g., Health Action Process Approach, (Schwarzer, 2008)]. Previous studies of photoprotection behaviour in student samples have reported positive relationships (Allom *et al*., 2013) as have adherence studies in other chronic conditions (Durand *et al*., 2018; Phillips, Cohen, Burns, Abrams, & Renninger, 2016). The development of habitual photoprotection could contribute to the negative association between age at the time of diagnosis and adherence in adults. Behaviour changes started in childhood might encourage the development of habit and greater acceptance of the necessity of photoprotection. As photoprotection is performed daily, this facilitates habit formation where repetition of behaviours in the same context is key (Lally, van Jaarsveld, Potts, & Wardle, 2010). Automatic processes might be especially important, as photoprotection is likely to be triggered by environmental cues such as sunlight (Andersen *et al*., 2016). Future research needs to investigate longitudinally the relative importance of beliefs versus habitual drivers in photoprotection maintenance.

Consistent with findings in the wider literature, illness‐ and treatment‐related cognitions, especially necessity beliefs, were related to adherence in XP. This supports the utility of treatment beliefs to explain variation in preventative behavioural regimes, as well as adherence to prescribed medicines (Foot, La Caze, Gujral, & Cottrell, 2016). Few studies have investigated the relationship between perceptions of skin cancer and protection activities. Cameron (2008) studied illness risk representations of skin cancer in a student sample and found that beliefs in weak treatment control were associated with better protection. We found personal rather than treatment control to be more important, perhaps due to robust measurement of photoprotection and control of clinical confounders. Those with greater concerns about photoprotection activities have a stronger tendency to avoid going outside during the day. It seems likely that greater concerns about wearing photoprotective clothing/sunscreen may tip the balance towards staying indoors, which may be a more acceptable way of coping for some individuals. It is noteworthy that having a negative emotional representation of XP was associated with staying indoors. Given the cross‐sectional design, we do not know whether this is driven by photoprotection preferences or whether it is a consequence of emotional distress. The burden of living with XP includes stigma related to changes in appearance, constant UVR monitoring, and worries about skin cancer (Anderson *et al*., 2017; Morgan *et al*., 2019). Further research needs to investigate the prevalence of emotional distress associated with photoprotection in XP to ascertain if there is something intrinsic about photoprotection that is detrimental to well‐being.

Staying indoors is not actively encouraged by the clinical teams as it is not a feasible option for all and contrary to the team’s wish to promote quality of life within the confines of extreme photoprotection. This is reflected in the findings as participants protected themselves more frequently by using sunscreen and clothing. In addition, avoidance of outdoors is more influenced by the context of people’s lives and demands of work, school, and other external constraints. This is supported by the finding that little variance was explained in the final model of avoidance of going outside, suggesting that it has different determinants not measured here (e.g., occupation). The importance of these contextual factors in adherence is emphasized by a recent OECD report (Khan & Socha‐Dietrich, 2018). If the study were to be replicated in non‐Western low‐ and middle‐income countries, their different health care systems and sociocultural environments should be considered.

Adherence in the cared‐for sample was higher than in the adults, which could be explained by the impact of having somebody monitoring and helping patients to meet their health needs. Whilst this could potentially be a reporting bias, a number of psychological correlates exhibited by caregivers are consistent with those found to be more favourable to adherence to photoprotection in other populations including higher self‐efficacy (Craciun, Schüz, Lippke, & Schwarzer, 2012), stronger intention (Starfelt Sutton & White, 2016), and greater automaticity (Allom *et al*., 2013). Although the cared‐for sample reported higher photoprotection, social support, in terms of both the perceived level and quality of support, in the whole sample was not related to adherence. Given the growing literature on the importance of perceived social support in treatment adherence (DiMatteo, 2004; Scheurer, Choudhry, Swanton, Matlin, & Shrank, 2012) and the complexity of the interactions between provider and recipient, future qualitative research is required to explore how social support influences photoprotection activities from the perspective of the XP patient.

### Limitations and future research

There are a number of limitations to the present study. Naturally, the cross‐sectional design means that causality cannot be ascertained and we are investigating the relationship between current psychological factors and *past* rather than *future* behaviour. Further prospective studies are recommended to investigate change in variables over time, although recent longitudinal photodermatological research in a healthy population (Thieden, Holm‐Schou, Philipsen, Heydenreich, & Wulf, 2019) and data from a related N‐of‐1 study carried out over 50 days suggest that UVR exposure and photoprotection are relatively stable within individuals (Sainsbury *et al.,* 2018). Due to the modest overall variance explained in photoprotection, we also speculate whether the questionnaire missed other psychological correlates, such as those which have since been identified by related qualitative research (e.g., resistance to XP identity; Morgan *et al.,* 2019). Concerns about questionnaire length voiced by the PPI panel were a contributing factor to the decision to limit constructs measured and use shortened versions. Another limitation is that adherence is likely to be lower than observed since we used a self‐report measure. A validation of our bespoke adherence tool against UVR dosimetry over a three‐week period indicated that whilst those reporting suboptimal adherence typically did not protect well against UVR, there was greater variability in UVR protection for those self‐reporting high adherence (Canfield *et al*., 2018).

Due to the rarity of the condition, it was necessary to recruit from a number of different countries. Whilst this may enhance the generalizability of our findings, this relies on there being no differences in the strength of the association between predictor variables across countries (i.e., no country by predictor interaction). The sample size limited the possibility to analyse photoprotection differences between countries. Given the challenges of recruiting participants in rare disease research (Kwakkenbos *et al*., 2013; Sainsbury, Walburn, Araujo‐Soares, & Weinman, 2018), we had to extend the data collection period beyond the summer months (May to December 2016) and seasonal differences might have had an influence on some responses. Despite these limitations, this is the only survey that has collected internationally comparable quantitative data in people diagnosed with XP. It is unlikely that larger studies are feasible in the target countries due to the response rate of 57% of those invited already being included here.

### Implications for intervention design

A number of modifiable psychological factors associated with photoprotection were identified that may be amenable to intervention. We would recommend that an intervention incorporates content to strengthen necessity by exploring specific beliefs that underpin doubts and resolving misunderstandings. Similar techniques have been effective in other chronic conditions (Broadbent, Ellis, Thomas, Gamble, & Petrie, 2009; O'Carroll, Chambers, Dennis, Sudlow, & Johnston, 2013). Communicating the cumulative nature of UVR damage would be particularly important. To maximize efficacy, resultant elevation of perceived threat should be accompanied by content to emphasize response efficacy (Tannenbaum *et al*., 2015). Promotion of habitual photoprotection by anchoring new behaviours to existing habits, creating environmental cues, and repeating in the same context should be included (Gardner, 2015). The reduced photoprotection on cloudy days reported here has serious implications for patients since UVR damage is still incurred and cloudy days are frequent in Western Europe. Therefore, we suggest that content related to psychological drivers are linked to weather conditions (e.g., emphasizing that external prompts to protect are more important in cloudy weather when there are fewer natural cues, such as sunlight). Given the complex nature of photoprotection, interventions to improve photoprotection will need to be tailored both to the individual’s pattern of drivers and to the particular photoprotection activity, since avoiding going outside and photoprotection whilst outside are influenced by different factors.

## Funding

This study/project is funded by the National Institute for Health Research (NIHR) [Programme Grant for Applied Research Scheme (RP‐PG‐1212 20009)]. The views expressed are those of the author(s) and not necessarily those of the NIHR or the Department of Health and Social Care.

## Conflicts of interest

All authors declare no conflict of interest**.**

## Author contributions

As joint first authors, JWa and MC were equal contributors and all authors reviewed and commented on the manuscript. All authors (excluding MC) were involved in the study design and development of the questionnaire. JW and LF were involved in the translation of questionnaires and all data collection. MC and SN conducted the analysis assisted by NM.

## Supporting information


**Table S1.** Univariate ordinal logistic regression of photoprotection practices on demographic, clinical and psychological variables.

## References

[bjhp12375-bib-0001] Allom, V. , Mullan, B. , & Sebastian, J. (2013). Closing the intention–behaviour gap for sunscreen use and sun protection behaviours. Psychology and Health, 28(5), 477–494. 10.1080/08870446.2012.745935 23252669

[bjhp12375-bib-0002] Andersen, P. A. , Buller, D. B. , Walkosz, B. J. , Scott, M. D. , Beck, L. , Liu, X. , … Eye, R. (2016). Environmental variables associated with vacationers' sun protection at warm weather resorts in North America. Environmental Research, 146, 200–206. 10.1016/j.envres.2015.12.034. 26775001 PMC13159384

[bjhp12375-bib-0003] Anderson, R. , Walburn, J. , & Morgan, M. (2017). Experiences of stigma over the lifetime of people with xeroderma pigmentosum: A qualitative interview study in the United Kingdom. Journal of Health Psychology. Advance online publication. 10.1177/1359105317714643 28810476

[bjhp12375-bib-0004] Araújo‐Soares, V. , Hankonen, N. , Presseau, J. , Rodrigues, A. , & Sniehotta, F. F. (2019). Developing behavior change interventions for self‐management in chronic illness: An integrative overview. European Psychologist, 24(1), 7 10.1027/1016-9040/a000330. 31496632 PMC6727632

[bjhp12375-bib-0005] Bradford, P. T. , Goldstein, A. M. , Tamura, D. , Khan, S. G. , Ueda, T. , Boyle, J. , … Kraemer, K. H. (2011). Cancer and neurologic degeneration in xeroderma pigmentosum: long term follow‐up characterises the role of DNA repair. Journal of Medical Genetics, 48, 168–176. 10.1136/jmg.2010.083022 21097776 PMC3235003

[bjhp12375-bib-0006] Bränström, R. , Kasparian, N. A. , Chang, Y.‐M. , Affleck, P. , Tibben, A. , Aspinwall, L. G. , … Bergman, W. (2010). Predictors of sun protection behaviors and severe sunburn in an international online study. Cancer Epidemiology and Prevention Biomarkers, 19, 2199–2210. 10.1158/1055-9965.EPI-10-0196 PMC3672402 20643826

[bjhp12375-bib-0007] Broadbent, E. , Donkin, L. , & Stroh, J. C. (2011). Illness and treatment perceptions are associated with adherence to medications, diet, and exercise in diabetic patients. Diabetes Care, 34(2), 338–340. 10.2337/dc10-1779 21270191 PMC3024345

[bjhp12375-bib-0008] Broadbent, E. , Ellis, C. , Thomas, J. , Gamble, G. , & Petrie, K. (2009). Further development of an illness perception intervention for myocardial infarction patients: A randomized controlled trial. Journal of Psychosomatic Research, 67(1), 17–23. 10.1016/j.jpsychores.2008.12.001 19539814

[bjhp12375-bib-0009] Broadbent, E. , Petrie, K. J. , Main, J. , & Weinman, J. (2006). The brief illness perception questionnaire. Journal of Psychosomatic Research, 60(6), 631–637. 10.1016/j.jpsychores.2005.10.020 16731240

[bjhp12375-bib-0010] Cameron, L. D. (2008). Illness risk representations and motivations to engage in protective behavior: The case of skin cancer risk. Psychology and Health, 23(1), 91–112. 10.1080/14768320701342383 25159909

[bjhp12375-bib-0011] Cane, J. , O’Connor, D. , & Michie, S. (2012). Validation of the theoretical domains framework for use in behaviour change and implementation research. Implementation Science, 7(1), 37 10.1186/1748-5908-7-37 22530986 PMC3483008

[bjhp12375-bib-0012] Canfield, M. , Norton, S. , Walburn, J. , Morrison‐Bowen, N. , Araujo‐Soares, V. , Sainsbury, K. , Sarkany, B. , & Weinman, J. (2018). Adherence to face photoprotection behaviours in xerederma pigmentosum patients: initial validation of a new measure. 10.31234/osf.io/6g7ba 31596975

[bjhp12375-bib-0013] Craciun, C. , Schüz, N. , Lippke, S. , & Schwarzer, R. (2012). A mediator model of sunscreen use: A longitudinal analysis of social‐cognitive predictors and mediators. International Journal of Behavioral Medicine, 19(1), 65–72. 10.1007/s12529-011-9153x 21394444

[bjhp12375-bib-0014] DiMatteo, M. R. (2004). Social support and patient adherence to medical treatment: A meta‐analysis. Health Psychology, 23(2), 207 10.1037/0278-6133.23.2.207 15008666

[bjhp12375-bib-0015] Durand, H. , Hayes, P. , Harhen, B. , Conneely, A. , Finn, D. P. , Casey, M. , … Molloy, G. J. (2018). Medication adherence for resistant hypertension: Assessing theoretical predictors of adherence using direct and indirect adherence measures. British Journal of Health Psychology, 23(4), 949–966. 10.1111/bjhp.12332 30014548

[bjhp12375-bib-0016] Eldredge, L. K. B. , Markham, C. M. , Kok, G. , Ruiter, R. A. , & Parcel, G. S. (2016). Planning health promotion programs: An intervention mapping approach. Hoboken, NJ: John Wiley & Sons.

[bjhp12375-bib-0017] Foot, H. , La Caze, A. , Gujral, G. , & Cottrell, N. (2016). The necessity–concerns framework predicts adherence to medication in multiple illness conditions: A meta‐analysis. Patient Education and Counseling, 99, 706–717. 10.1016/j.pec.2015.11.004 26613666

[bjhp12375-bib-0018] Francis, J. , Eccles, M. P. , Johnston, M. , Walker, A. , Grimshaw, J. , Foy, R. , … Bonetti, D. (2004). Constructing questionnaires based on the theory of planned behaviour: A manual for health services researchers: Centre for Health Services Research, University of Newcastle upon Tyne.

[bjhp12375-bib-0019] Gardner, B. (2015). A review and analysis of the use of ‘habit’ in understanding, predicting and influencing health‐related behaviour. Health Psychology Review, 9(3), 277–295. 10.1080/17437199.2013.876238 25207647 PMC4566897

[bjhp12375-bib-0020] Gardner, B. , Abraham, C. , Lally, P. , & de Bruijn, G.‐J. (2012). Towards parsimony in habit measurement: Testing the convergent and predictive validity of an automaticity subscale of the Self‐Report Habit Index. International Journal of Behavioral Nutrition and Physical Activity, 9(1), 102 10.1186/1479-5868-9-102 22935297 PMC3552971

[bjhp12375-bib-0021] Good, A. , & Abraham, C. (2011). Can the effectiveness of health promotion campaigns be improved using self‐efficacy and self‐affirmation interventions? An analysis of sun protection messages. Psychology and Health, 26(7), 799–818. 10.1080/08870446.2010.495157 21432728

[bjhp12375-bib-0022] Hagger, M. S. , Koch, S. , Chatzisarantis, N. L. , & Orbell, S. (2017). The common sense model of self‐regulation: Meta‐analysis and test of a process model. Psychological Bulletin, 143(11), 1117 10.1037/bul0000118 28805401

[bjhp12375-bib-0023] Horne, R. , Chapman, S. C. , Parham, R. , Freemantle, N. , Forbes, A. , & Cooper, V. (2013). Understanding patients’ adherence‐related beliefs about medicines prescribed for long‐term conditions: A meta‐analytic review of the Necessity‐Concerns Framework. PLoS ONE, 8(12), e80633 10.1371/journal.pone.0080633 24312488 PMC3846635

[bjhp12375-bib-0024] Horne, R. , Weinman, J. , & Hankins, M. (1999). The beliefs about medicines questionnaire: The development and evaluation of a new method for assessing the cognitive representation of medication. Psychology and Health, 14(1), 1–24. 10.1080/08870449908407311

[bjhp12375-bib-0025] Kardas, P. , Lewek, P. , & Matyjaszczyk, M. (2013). Determinants of patient adherence: a review of systematic reviews. Frontiers in Pharmacology, 4, 91 10.3389/fphar.2013.00091 23898295 PMC3722478

[bjhp12375-bib-0026] Khan, R. , & Socha‐Dietrich, K. (2018). Investing in medication adherence improves health outcomes and health system efficiency. 10.1787/8178962c-en

[bjhp12375-bib-0027] Kleijer, W. J. , Laugel, V. , Berneburg, M. , Nardo, T. , Fawcett, H. , Gratchev, A. , … Lehmann, A. R. (2008). Incidence of DNA repair deficiency disorders in western Europe: Xeroderma pigmentosum, Cockayne syndrome and trichothiodystrophy. DNA Repair, 7, 744–750. 10.1016/j.dnarep.2008.01.014 18329345

[bjhp12375-bib-0028] Kwakkenbos, L. , Jewett, L. R. , Baron, M. , Bartlett, S. J. , Furst, D. , Gottesman, K. , … Mouthon, L. (2013). The Scleroderma Patient‐centered Intervention Network (SPIN) Cohort: protocol for a cohort multiple randomised controlled trial (cmRCT) design to support trials of psychosocial and rehabilitation interventions in a rare disease context. British Medical Journal Open, 3(8), e003563 10.1136/bmjopen-2013-003563 PMC3740254 23929922

[bjhp12375-bib-0029] Lally, P. , van Jaarsveld, C. H. M. , Potts, H. W. W. , & Wardle, J. (2010). How are habits formed: Modelling habit formation in the real world. European Journal of Social Psychology, 40, 998–1009. 10.1002/ejsp.674.

[bjhp12375-bib-0030] Lehmann, A. R. , McGibbon, D. , & Stefanini, M. (2011). Xeroderma pigmentosum. Orphanet Journal of Rare Diseases, 6(1), 70 10.1186/1750-1172-6-70 22044607 PMC3221642

[bjhp12375-bib-0031] Leventhal, H. , Phillips, L. A. , & Burns, E. (2016). The Common‐Sense Model of Self‐Regulation (CSM): A dynamic framework for understanding illness self‐management. Journal of Behavioral Medicine, 39, 935–946. 10.1007/s10865-016-9782-2 27515801

[bjhp12375-bib-0032] Marteau, T. M. , Hollands, G. J. , & Fletcher, P. C. (2012). Changing human behavior to prevent disease: The importance of targeting automatic processes. Science, 337, 1492–1495. https://doi.org/10.1126.science.1226918 22997327 10.1126/science.1226918

[bjhp12375-bib-0033] Michie, S. , van Stralen, M. M. , & West, R. (2011). The behaviour change wheel: A new method for characterising and designing behaviour change interventions. Implementation Science, 6(1), 42 10.1186/1748-5908-6-42 21513547 PMC3096582

[bjhp12375-bib-0034] Nahar, V. K. , Ford, M. A. , Brodell, R. T. , Boyas, J. F. , Jacks, S. K. , Biviji‐Sharma, R. , … Bass, M. A. (2016). Skin cancer prevention practices among malignant melanoma survivors: A systematic review. Journal of Cancer Research and Clinical Oncology, 142, 1273–1283. 10.1007/s00432-015-2086-z 26642962 PMC11819181

[bjhp12375-bib-0035] Morgan, M. , Anderson, R. , Walburn, J. , Weinman, J. , & Sarkany, R. (2019). The influence of perceived medical risks and psychosocial concerns on photoprotection behaviours among adults with xeroderma pigmentosum: a qualitative interview study in the UK. BMJ Open, 9(2), e024445 10.1136/bmjopen-2018-024445 PMC6377541 30782905

[bjhp12375-bib-0036] O'Carroll, R. E. , Chambers, J. A. , Dennis, M. , Sudlow, C. , & Johnston, M. (2013). Improving adherence to medication in stroke survivors: A pilot randomised controlled trial. Annals of Behavioral Medicine, 46(3), 358–368. 10.1007/s12160-013-9515-5 23670112

[bjhp12375-bib-0037] Oo, C. , & Rusch, L. M. (2016). A personal perspective of orphan drug development for rare diseases: A golden opportunity or an unsustainable future? The Journal of Clinical Pharmacology, 56(3), 257–259. 10.1002/jcph.599 26211513

[bjhp12375-bib-0038] Pettigrew, S. , Jongenelis, M. , Strickland, M. , Minto, C. , Slevin, T. , Jalleh, G. , & Lin, C. (2016). Predictors of sun protection behaviours and sunburn among Australian adolescents. BMC Public Health, 16(1), 565 10.1186/s12889-016-3197-4 27411518 PMC4944266

[bjhp12375-bib-0039] Phillips, L. A. , Cohen, J. , Burns, E. , Abrams, J. , & Renninger, S. (2016). Self‐management of chronic illness: The role of ‘habit’ versus reflective factors in exercise and medication adherence. Journal of Behavioral Medicine, 39, 1076–1091. 10.1007/s10865-016-9732-z 26980098

[bjhp12375-bib-0040] Sainsbury, K. , Vieira, R. , Walburn, J. , Sniehotta, F. F. , Sarkany, R. , Weinman, J. , & Araujo‐Soares, V. (2018). Understanding and predicting a complex behavior using n‐of‐1 methods: Photoprotection in xeroderma pigmentosum. Health Psychology, 37(12), 1145 10.1037/hea0000673 30221970

[bjhp12375-bib-0042] Sainsbury, K. , Walburn, J. , Araujo‐Soares, V. , & Weinman, J. (2018). Challenges and proposed solutions for formative research to inform systematic intervention development in rare and unstudied conditions: The case example of Xeroderma Pigmentosum. British Journal of Health Psychology, 23(2), 229–237. 10.1111/bjhp.12287 29265545 PMC5901402

[bjhp12375-bib-0041] Sarason, I. G. , Levine, H. M. , Basham, R. B. , & Sarason, B. R. (1983). Assessing social support: The social support questionnaire. Journal of Personality and Social Psychology, 44(1), 127 10.1037/0022-3514.44.1.127

[bjhp12375-bib-0043] Scheurer, D. , Choudhry, N. , Swanton, K. A. , Matlin, O. , & Shrank, W. (2012). Association between different types of social support and medication adherence. The American Journal of Managed Care, 18(12), e461–467. 23286676

[bjhp12375-bib-0044] Schwarzer, R. (2008). Modeling health behavior change: How to predict and modify the adoption and maintenance of health behaviors. Applied Psychology, 57(1), 1–29. https://doi.org/10.111.j.1464-0597.2007.00325.x

[bjhp12375-bib-0045] Sethi, M. , Lehmann, A. , Fawcett, H. , Stefanini, M. , Jaspers, N. , Mullard, K. , … Sarkany, R. (2013). Patients with xeroderma pigmentosum complementation groups C, E and V do not have abnormal sunburn reactions. British Journal of Dermatology, 169, 1279–1287. 10.1111/bjd.12523 23889214

[bjhp12375-bib-0046] Starfelt Sutton, L. C. , & White, K. M. (2016). Predicting sun‐protective intentions and behaviours using the theory of planned behaviour: a systematic review and meta‐analysis. Psychology and Health, 31(11), 1272–1292. 10.1080/08870446.2016.1204449 27334551

[bjhp12375-bib-0047] Tamura, D. , DiGiovanna, J. J. , Khan, S. G. , & Kraemer, K. H. (2014). Living with xeroderma pigmentosum: Comprehensive photoprotection for highly photosensitive patients. Photodermatology, Photoimmunology and Photomedicine, 30(2–3), 146–152. 10.1111/phpp.12108 PMC6334764 24417420

[bjhp12375-bib-0048] Tannenbaum, M. B. , Hepler, J. , Zimmerman, R. S. , Saul, L. , Jacobs, S. , Wilson, K. , & Albarracín, D. (2015). Appealing to fear: A meta‐analysis of fear appeal effectiveness and theories. Psychological Bulletin, 141, 1178 10.1037/a0039729. 26501228 PMC5789790

[bjhp12375-bib-0049] Thieden, E. , Holm‐Schou, A.‐S. S. , Philipsen, P. A. , Heydenreich, J. , & Wulf, H. C. (2019). Adult UVR exposure changes with life stage–a 14‐year follow‐up study using personal electronic UVR dosimeters. Photochemical and Photobiological Sciences, 18, 467–476. 10.1039/C8PP00365C 30511738

[bjhp12375-bib-0050] Verplanken, B. , & Orbell, S. (2003). Reflections on past behavior: A self‐report index of habit strength. Journal of Applied Social Psychology, 33, 1313–1330. 10.1111/j.1559-1816.2003.tb01951.x

[bjhp12375-bib-0051] Walburn, J. , Sarkany, R. , Norton, S. , Foster, L. , Morgan, M. , Sainsbury, K. , & Heydenreich, J. (2017). An investigation of the predictors of photoprotection and UVR dose to the face in patients with XP: A protocol using observational mixed methods. BMJ Open, 7(8), e018364 10.1136/bmjopen-2017-018364. PMC5724163 28827277

[bjhp12375-bib-0052] WHO (2018). Process of translation and adaptation of instruments. Retrieved from http://www.who.int/substance_abuse/research_tools/translation/en/

[bjhp12375-bib-0053] Wu, Y. P. , Aspinwall, L. G. , Conn, B. M. , Stump, T. , Grahmann, B. , & Leachman, S. A. (2016). A systematic review of interventions to improve adherence to melanoma preventive behaviors for individuals at elevated risk. Preventive Medicine, 88, 153–167. 10.1016/j.ypmed.2016.04.010 27090434 PMC4902721

