# Supplementary material for: Psychological correlates of adherence to photoprotection in a rare disease: International survey of people with Xeroderma Pigmentosum
Source: Br J Health Psychol. 2019 Jun 10;24(3):668–86. doi: 10.1111/bjhp.12375 (PMC6772157; doi:10.1111/bjhp.12375)
Supplement: Supplementary file 1 — Table S1. Univariate ordinal logistic regression of photoprotection practices on demographic, clinical and psychological variables. [file BJHP-24-668-s001.docx]

**Supplementary Table 1.** Univariate Ordinal Logistic Regression of Photoprotection Practices on Demographic, Clinical and Psychological Variables

|  | ***Adherence to face photoprotection***  ***OR (95% CI)*** | ***p*** | ***Adherence to body photoprotection***  ***OR (95% CI)*** | ***p*** | ***Avoidance of going***  ***outside***  ***OR (95% CI)*** | ***p*** | |
| --- | --- | --- | --- | --- | --- | --- | --- |
| ***Demographic variables*** |  |  |  |  |  | |  |
| Female gender | .81 (.44, 1.49) | .507 | .85 (.45, 1.46) | .495 | 1.41 (.81, 2.45) | | .222 |
| Age* | **.61 (.44, .83)** | **.002** | **.68 (.50, .92)** | **.013** | .80 (.60, 1.05) | | .118 |
| Fitzpatrick skin type IV/V | .73 (.39, 1.37) | .332 | .66 (.37, 1.89) | .167 | .58 (.33, 1.01) | | .053 |
| Education level* | **.41 (.30, .57)** | **<.001** | **.45 (.33, .62)** | **<.001** | 1.64 (.84, 1.45) | | .489 |
| ***Clinical variables*** | | | | | | | |
| Age at diagnosis* | **.59 (.43, .81)** | **.001** | **.74 (.55, .99)** | **.046** | 1.01 (.76, 1.34) | | .932 |
| Propensity to not burn | .65 (.34, 1.24) | .195 | .62(.33, 1.58) | .135 | .86 (.48, 1.56) | | .629 |
| History of skin cancer | .55 (.30, 1.02) | .058 | .59 (.33, 1.07) | .081 | .94 (.54, 1.63) | | .831 |
| XP related problems with hearing/speaking/walking | 1.13 (.56, 2.25) | .737 | 1.05 (.54, 2.02) | .887 | .82 (.42, 1.58) | | .555 |
| Eyesight problems | 1.40 (.71, 2.75) | .333 | 1.43 (.74, 2.76) | .280 | 1.09 (.6, 2.00) | | .773 |
| ***Psychological variables*** | | | | | | | |
| XP Perceptions | | | | | | | |
| Consequences | 1.31 (.96, 1.80) | .088 | **1.49 (1.06, 2.01)** | **.009** | **1.86(1.39, 2.50)** | | **<.001** |
| Timeline | .73 (.48, 1.10) | .141 | .74 (.50, 1.09) | .135 | 1.01 (.74, 1.34) | | .997 |
| Personal control of XP | **1.70 (1.25, 2.30)** | **.001** | **1.65 (1.22, 2.23)** | **.001** | .88 (.67, 1.16) | | .368 |
| Photoprotection control of XP | **1.85 (1.40, 2.45)** | **<.001** | **1.63 (1.23, 2.16)** | **.001** | .92(.70, 1.22) | | .570 |
| Treatment control | .86 (.62, 1.20) | .382 | .84 (.61, 1.15) | .287 | .71 (.62, 1.09) | | .182 |
| Identity | .85 (.62, 1.16) | .309 | .96 (.71, 1.31) | .817 | 1.12 (.85, 1.48) | | .416 |
| Illness concern | .87 (.63, 1.19) | .390 | 1.04 (.78, 1.39) | .784 | **1.55 (1.15, 2.08)** | | **.004** |
| Understanding | .74 (.54, 1.01) | .066 | **.72 (53, .97)** | **.033** | .88 (.67, 1.17) | | .409 |
| Emotional representation | 1.21 (.90, 1.64) | .208 | 1.29 (.96, 1.74) | .088 | **1.41 (1.06, 1.88)** | | **.020** |
| Beliefs about Photoprotection |  |  |  |  |  | |  |
| Necessity | **2.45 (1.76, 3.42)** | **<.001** | **2.73 (1.93, 3.86)** | **<.001** | 1.24 (.93, 1.65) | | .141 |
| Concern | .97 (.71, 1.31) | .854 | 1.16 (.87, 1.55) | .304 | **1.61 (1.21, 2.15)** | | **.001** |
| Intention to photoprotect**/ avoid going outside | **1.76 (1.27, 2.44)** | **.001** | **1.56 (1.14, 2.12)** | **.005** | 1.07 (.85, 1.34) | | .547 |
| Self-efficacy to photoprotect**/ avoid going outside | **1.66 (1.21, 2.29)** | **.002** | **1.56 (1.47, 2.13)** | **.005** | **2.25 (1.66, 3.4)** | | **<.001** |
| Automaticity for photoprotection**/ avoid going outside | **2.38 (1.67, 3.38)** | **<.001** | **2.36 (1.69, 3.31)** | **<.001** | **2.48 (1.82, 3.38)** | | **<.001** |
| Social support | 1.06 (.79, 1.42) | .692 | 1.08 (.83, 1.43) | .491 | .87 (.69, 1.15) | | .338 |
| Effectiveness of photoprotection** | **2.18 (1.53, 3.10)** | **<.001** | **2.08 (1.48, 2.92)** | **<.001** | 1.10 (.82, 1.45) | | .554 |

Note: Bold indicates statistically significant associations.

*p values for adult sample only on face/body photoprotection: age = <.001; <.001; age diagnosed = .031; <.001; educational level = .811; .198.

**wearing sunscreen, clothing when outside
